# Uncoupling of oxidative phosphorylation and ATP synthase reversal within the hyperthermic heart

**DOI:** 10.14814/phy2.12138

**Published:** 2014-09-28

**Authors:** Amelia Power, Nicholas Pearson, Toan Pham, Carlos Cheung, Anthony Phillips, Anthony Hickey

**Affiliations:** 1 School of Biological Sciences, Faculty of Science, University of Auckland, Auckland, New Zealand; 2 Department of Physiology, Faculty of Medical and Health Sciences, University of Auckland, Auckland, New Zealand; 3 Department of Surgery, Faculty of Medical and Health Sciences, University of Auckland, Auckland, New Zealand; 4 Maurice Wilkins Center, University of Auckland, Auckland, New Zealand

**Keywords:** ATP synthesis, hyperthermia, mitochondrial membrane potential, mitochondrial respiration

## Abstract

Heart failure is a common cause of death with hyperthermia, and the exact cause of hyperthermic heart failure appears elusive. We hypothesize that the energy supply (ATP) of the heart may become impaired due to increased inner‐mitochondrial membrane permeability and inefficient oxidative phosphorylation (OXPHOS). Therefore, we assessed isolated working heart and mitochondrial function. Ex vivo working rat hearts were perfused between 37 and 43.5°C and showed break points in all functional parameters at ~40.5°C. Mitochondrial high‐resolution respirometry coupled to fluorometry was employed to determine the effects of hyperthermia on OXPHOS and mitochondrial membrane potential (Δ*Ψ*) in vitro using a comprehensive metabolic substrate complement with isolated mitochondria. Relative to 37 and 40°C, 43°C elevated Leak O_2_ flux and depressed OXPHOS O_2_ flux and ∆*Ψ*. Measurement of steady‐state ATP production from mitochondria revealed decreased ATP synthesis capacity, and a negative steady‐state P:O ratio at 43°C. This approach offers a more powerful analysis of the effects of temperature on OXPHOS that cannot be measured using simple measures such as the traditional respiratory control ratio (RCR) or P:O ratio, which, respectively, can only approach 1 or 0 with inner‐membrane failure. At 40°C there was only a slight enhancement of the Leak O_2_ flux and this did not significantly affect ATP production rate. Therefore, during mild hyperthermia (40°C) there is no enhancement of ATP supply by mitochondria, to accompany increasing cardiac energy demands, while between this and critical hyperthermia (43°C), mitochondria become net consumers of ATP. This consumption may contribute to cardiac failure or permanent damage during severe hyperthermia.

## Abstract (precis)

e12138

Intact cardiac function is crucial, and is particularly important for mediating thermoregulation in hyperthermic states. However, the heart's aerobic metabolism is also thermally sensitive, and may become limited at extreme physiological temperatures. We have shown that at 43°C there is a loss of mitochondrial membrane potential, and this subsequently drives a reversal of the mitochondrial the F_1_F_0_‐ATP synthase where it becomes a net consumer of ATP.

## Introduction

It is known that high core temperatures in humans (usually considered above 40°C) can result in tissue damage, organ failure, and death (Marshall 2010). Cardiovascular dysfunction often occurs early in multiorgan failure during severe heat stroke making it central to hyperthermia‐mediated morbidity or death (Bouchama et al. 2007). The heart is important in normal thermoregulation and so physiological studies have previously characterized the cardiovascular response to hyperthermia in the context of rest, exercise, and heart disease (Whittow 1965; Cui et al. 2005; Trinity et al. 2010). At the subcellular level there is reportedly an increase in cardiac mitochondrial inner‐membrane permeability during severe hyperthermia (42°C), which may in turn compromise adenosine triphosphate (ATP) supply (Qian et al. 2004; Zukiene et al. 2010; Nauciene et al. 2012). It is therefore likely that the cardiovascular dysfunction associated with increasing hyperthermia may be due to a mismatch between energy supply and demand.

Within ectotherms (cold bodied animals, e.g., fishes, reptiles, etc.), cardiac metabolism must be able to accommodate broad variations in temperature during the diurnal cycle or with seasons. So while cardiac function ultimately collapses at high temperatures, some plasticity to temperature is observed (Iftikar et al. 2010; Galli and Richards 2012; Iftikar and Hickey 2013). Conversely, organs within homeothermic mammals are highly constrained to function at relatively high temperatures (35–39°C in marsupials to bovids, and 39–42°C in aves). A surprisingly small rise from basal temperatures results in cardiac dysfunction (Haupt and Rackow 1983), so that the mammalian heart can be considered to sit on the cusp of hyperthermic heart failure.

Homeotherms have greater sustained cardiac demands in order to support O_2_ delivery to highly metabolic tissues. With hyperthermia cardiac demands increase to elevate peripheral perfusion for convective heat dissipation and to compensate for increased metabolic rates of reaction throughout the body. Hyperthermia‐mediated increases in cutaneous vasodilation are accompanied by elevated cardiac outputs (CO) to maintain arterial pressure (Lenhardt et al. 2008), and this has been reported in human subjects to drive resting CO from ~5 L min^−1^ at thermoneutrality, up to 13 L min^−1^ (at 38.8°C)(Rowell et al. 1969).

Increased cardiac work mediated by hyperthermia might therefore progressively compromise maximal physical activity during exercise. However, mild hyperthermia (increased core temperature by 0.8°C) can enhance cardiovascular function (Trinity et al. 2010), and trained athletes can reach core body temperatures of 40.9°C with no detectable impacts on time trial performances (Ely et al. 2009). Therefore, core body temperatures approaching 41°C can be tolerated.

Pharmacological‐induced malignant hyperthermia, and fevers resulting from infection, extreme environmental temperature exposure, and extreme exercise can elevate temperatures above 43°C, and this appears to be detrimental and can even be fatal (Vertree et al. 2002; Lenhardt et al. 2008). Moreover those with heart diseases are more vulnerable to heat stress (Semenza et al. 1999), and this could be even more pronounced in patients with cardiac deficiencies resulting from mitochondrial oxidative phosphorylation (OXPHOS) system defects (Lemieux et al. 2011).

While hyperthermia‐mediated cardiovascular failure is well documented, the cellular mechanisms that limit cardiomyocyte function are less clear, particularly in the context of mitochondrial function. To contract, the heart requires ATP, of which 95% is provided by OXPHOS (Kolwicz et al. 2013). Mitochondria may be critical in hyperthermic heart failure (Abele et al. 2002; Iftikar et al. 2010). While isolated cardiac mitochondria are reported to show a decrease in stability above 41°C, and a consequential decrease in membrane potential (Δ*Ψ*) (Zukiene et al. 2007, 2010), most investigators have primarily used single substrates to supply electrons to complex I (CI) of the electron transport system (ETS). This likely underestimates the influence of temperature on the complete ETS. In addition, direct measures of ATP synthesis rates have not been made to confirm the impact of temperature on this key functional output. In vivo mitochondria are supported by multiple electron inputs predominantly at complexes I and II. In addition, more recent developments in respirometry and fluorometry from our laboratory now permit real‐time detection of mitochondrial membrane potential (Δ*Ψ*) and direct quantification of ATP synthesis rates simultaneously with measured respiration (Goo et al. 2013). This approach is expected to add important new knowledge about the role of cardiac mitochondria in hyperthermia.

In this study we measured cardiovascular function in Sprague–Dawley rat ex vivo working hearts and in vitro cardiac mitochondrial function from tissue samples. We conducted this work with multiple metabolic substrates that maximally activate respirational flux, to better represent mitochondrial function in hardworking hearts. Overall this work has now provided further new detailed insights into how OXPHOS efficiency is impacted by hyperthermia.

## Materials and Methods

### Experimental animals

Ten‐ to 13‐week‐old male Sprague–Dawley (SD) rats (*n* = 13; six for ex vivo and seven for in vitro experiments) were fed normal chow ad libitum after weaning. Rats were housed at 21°C on a 12‐h light/dark cycle. Experimental use of these animals was approved by the University of Auckland Ethics Committee. All chemicals were obtained from Sigma‐Aldrich (St. Louis, MO) unless specified.

### Temperature effects on cardiac function (ex vivo working rat heart)

SD rats (*n* = 6) were heparinized (1000 IU kg^−1^ i.v.) under isoflurane anesthesia before rapid excision of the hearts, which then underwent retrograde (Langendorff) perfusion with Krebs–Henseleit bicarbonate buffer (containing in mmol L^−1^; 4.7 KCl, 2.3 CaCl_2_, 1.2 MgSO_4_ 1.2 KH_2_PO_4_, 118 NaCl, 24 NaHCO_3_, and 11 glucose). Following retrograde perfusion, working‐mode perfusion was performed and cardiac function was assessed as described previously (Jullig et al. 2008). Heart rate (HR), intra‐left ventricular (LV) pressure (transducer model SP855, AD Instruments), aortic pressure (transducer model PX23XL, Stratham Gould), cardiac output (CO), and aortic flow (transducer model T206, Transonic) were recorded using a PowerLab (ADInstruments). LV pressure development and relaxation (+/− LV dP/d*t*) and stroke volume (SV) were further derived from these measures. Hearts were subjected to working‐mode perfusion (preload 10 cmH_2_O, afterload 82.8 mmHg) unpaced and allowed to reach a stable baseline for 10 min at 37°C. Temperature was increased in 0.5°C increments (held for ~2 min at each temperature) up to 43.5°C.

### Mitochondrial bioenergetics

#### Mitochondrial isolation

Freshly excised SD rat hearts (*n* = 7) were immediately immersed in ice‐cold saline until contractions ceased. The heart was dissected into 50 mg pieces and stored in Custodial® Histidine–Tryptophan–Ketoglutarate (HTK) preservation buffer (containing in mmol L^−1^; 15 NaCl, 9 KCl, 1 KH‐2‐ketoglutarate, 4 MgCl_2_**·**6H_2_O, 18 histidine**·**HCl**·**H_2_O, 180 histidine, 2 tryptophan, 30 mannitol, and 0.015 CaCl_2_**·**2H_2_O; Essential Pharmaceuticals, Ewing, NJ). HTK is a transplant preservation solution and suppresses ischemia‐induced acidosis (Ku et al. 1997) and preserves cardiac mitochondrial function, with respiration levels maintained for up to 11 h of storage (Gnaiger et al. 2000).

Mitochondria were isolated at 4°C using a differential centrifugation method modified from those described previously (Qian et al. 2004; Zukiene et al. 2010). Approximately 400 mg of cardiac tissue (right and left ventricle) was homogenized (Omni TH, Omni International) in 1 mL of ice‐cold mitochondrial respiration buffer (RB; containing in mmol L^−1^; 0.5 EGTA, 3 MgCl_2_.6H_2_O, 60 K‐lactobionate, 20 taurine, 10 KH_2_PO_4_, 20 HEPES, 110 sucrose, and 1 g L^−1^ of BSA [bovine serum albumin], pH 7.1). The homogenate was filtered and centrifuged at 68*g* for 10 min. The supernatant was then centrifuged at 10,000*g* for a further 10 min and the pellet was resuspended in 100 *μ*L of cold RB and stored concentrated on ice (approximately 50 mg mL^−1^). The mitochondrial protein concentration was determined using Biuret reagent against a BSA standard (10 mgmL^−1^) (Gornall et al. 1949).

#### Respirometry

Mitochondrial respirometry assays were performed using Oroboros Oxygraph‐2k™ respirometers (Oroboros Instruments, Innsbruck, Austria). The oxygraph comprises two closed 2 mL chambers with polarographic O_2_ sensors (Clark type), held at a constant temperature by inbuilt Peltier systems with temperature control to ±0.001°C. Mitochondria (0.125 mg mL^−1^) were added to each chamber of the oxygraph suspended in 2 mL of RB. Respiration was measured as weight‐specific O_2_ flux (pmol s^−1^ mg^−1^), calculated in real‐time using the negative time derivative of O_2_ concentration and recorded in *DatLab5* (Oroboros Instrument™). Mitochondrial assays were run in duplicate for each heart and temperature.

Two different mitochondrial respiration assay protocols were performed that allowed *simultaneous* fluorometric measurements of either ATP production or ∆*Ψ* within the oxygraph chamber (fluorometric methods described below). All assays measured flux through the mitochondrial OXPHOS system with both complex I (CI) and II (CII) substrates, which provide convergent electron supply into the Q‐cycle of the ETS (Gnaiger 2008). This permits respirational flux measurements that are more representative of that predicted to occur in vivo than CI respiration alone (Gnaiger 2011). Each assay protocol was repeated at normothermia (37°C), moderate hyperthermia (40°C), and critical hyperthermia (43°C). The quality of the mitochondrial preparation was determined by measuring the respiratory control ratio (RCR) with CI substrates at 37°C and was found to be 7.33 ± 1.85. The O_2_ sensors were calibrated with the RB equilibrated to atmospheric O_2_ using the O_2_ concentrations of 192.98 *μ*mol L^−1^ (37°C), 183.56 *μ*mol L^−1^ (40°C), and 175.32 *μ*mol L^−1^ (43°C) as calculated by *DatLab5* and accounting for a barometric pressure of 101 KPa and an O_2_ solubility factor of 0.92 in RB.

#### ATP assay

**Figure 1.**
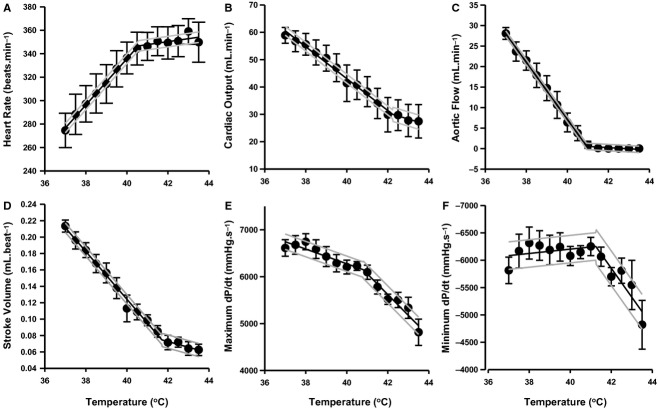
Cardiac function parameters measured in the ex vivo working heart with increasing temperature (*n* = 6). Whole hearts were perfused at 37°C for baseline measurements and then perfusate temperature was increased in 0.5°C (held for ~2 min) increments up to 43.5°C. Mean cardiac function was fitted with a segmented regression with 95% confidence lines and are shown as follows: heart rate (A, beats.min^−1^), cardiac output (B, mL min^−1^), aortic flow (C, mL min^−1^), stoke volume (D, mL beat^−1^), maximum rate of left ventricular (LV) pressure development (E, mmHg s^−1^) which is representative of cardiac contractility and minimum LV pressure relaxation (F, mmHg s^−1^). Each parameter is reported at a preload of 10 cmH_2_O.

Mitochondrial ATP production was determined in the oxygraph chamber by tracking changes in free extramitochondrial [Mg^2+^] using the fluorescent indicator, Magnesium Green (MgG; Molecular Probes^®^, Life Technologies, Carlsbad, CA), modified from Chinopoulos et al. (2009) and previously described by our laboratory (Fig. 1) (Goo et al. 2013). Fluorescence was measured with purpose‐built fluorometers described by Hickey et al. (2012) with filters adapted for a 503 nm excitation (Cyan LED) and 530 nm emission (Hickey et al. 2012). Mitochondria (0.125 mg mL^−1^) were added to 2 mL of RB containing MgG (5 *μ*mol L^−1^) in each chamber of the oxygraph. Steady‐state basal respiration (negligible) was reached before the addition of CI substrates; glutamate (10 mmol L^−1^), malate (2 mmol L^−1^), and pyruvate (10 mmol L^−1^) in order to determine the CI‐Leak (state 2) respiration. Succinate (10 mmol L^−1^) was added to measure the combined CI&CII‐Leak state. Finally, Mg^2+^‐free ADP (5 mmol L^−1^) was added to initiate CI&CII‐oxidative phosphorylation (OXPHOS; state 3). The assay was left to run into anoxia, after which, oligomycin (5 *μ*mol L^−1^) was added to block ATP hydrolysis by the F_1_F_0_‐ATP synthase (state 4), and this was taken as zero ATP production.

MgG fluorescence was recorded simultaneously with respiration using *DatLab5* and calibrated using the change in fluorescence before and after the addition of Mg^2+^‐free ADP (5 mmol L^−1^). During OXPHOS the conversion of ADP to ATP quenches the MgG fluorescence, as ATP has a higher affinity for Mg^2+^ than ADP. A correction factor was determined in independent assays using the ratio in fluorescence between equimolar (5 mmol L^−1^) concentrations of ATP and ADP in our respiration buffer containing Mg^2+^ (3 mmol L^−1^) and MgG (5 *μ*mol L^−1^). This correction factor was used to multiply the ADP signal and to calculate the ATP production rate (pmols s^−1^ mg^−1^) from the time derivative of the fluorescence signal.

#### Membrane potential assay

**Figure 2.**
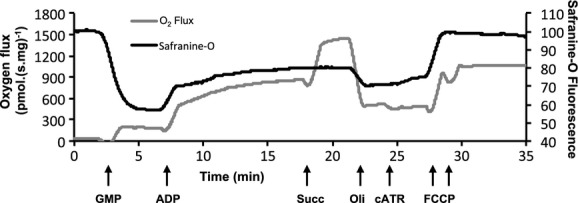
Simultaneous measurements of mitochondrial membrane potential and respiration. Mitochondrial respiration flux normalized to mass (gray line; pmolO_2._(s mg)^−1^) and safranine‐O fluorescence (black line; arbitrary fluorescence units) from a single oxygraph chamber at 37°C. The time derivative of the change in safranine‐O fluorescence was also calculated but is not shown for clarity. Arrows show sequential titrations of GMP (glutamate, malate, and pyruvate), ADP, Succ (succinate), Oli (oligomycin), cATR (carboxyatractyloside), and FCCP (carbonyl cyanide 4‐(trifluoromethoxy)‐phenylhydrazone).

A fluorometer with filters for an excitation and emission wavelength of 520 and 590 nm, respectively, was used to measure changes in safranine‐O fluorescence (Fig. 2). Mitochondria (0.125 mg mL^−1^) were added to each chamber in 2 mL of RB with safranine‐O (2 *μ*mol L^−1^). CI substrates glutamate (10 mmol L^−1^), pyruvate (10 mmol L^−1^), and malate (2 mmol L^−1^) were added to energize the mitochondria. In the absence of ADP, this state of respiration is CI‐Leak and allows the development of a high Δ*Ψ*. ADP (2.5 mmol L^−1^) was then added to initiate CI‐OXPHOS. CII substrate succinate (10 mmol L^−1^) was added to measure CI&CII‐OXPHOS and maximal activity of the ETS during OXPHOS. The inhibitors oligomycin (5 *μ*mol L^−1^) and carboxyatractyloside (cATR; 2 *μ*mol L^−1^) were titrated to induce CI&CII‐Leak, by blocking F_0_F_1_ATP‐synthase and adenosine nucleotide translocase (ANT), respectively. Finally, mitochondria were completely uncoupled by the addition of the uncoupling agent, FCCP (1 *μ*mol L^−1^).

Generally fluorescent probes used to follow Δ*Ψ* are reported as a relative fluorescence measure. The use of relative values requires normalization to a specific value (i.e., maximum or minimum Δ*Ψ*) and therefore information is lost. Most protocols use fluorophores such as JC‐1 and tetramethylrhodamine esters, which follow ratiometric properties and undergo spectral changes when following their movement into the mitochondrial matrix and/or stacking within mitochondria (Scaduto and Grotyohann 1999; Assayag et al. 2012). However, here we follow the cationic safranine‐O (unstacked) fluorescence in the media, which quenches on uptake and stacking within the mitochondrial matrix. The safranine‐O signal should therefore behave like other probes, such as TPP^+^, which are calibrated using the Nernst equation (Palmeira and Rolo 2012). While Δ*Ψ* determined using safranine‐O can be calibrated through K^+^ titrations in the presence of valinomycin (Figueira et al. 2012), our physiological RB contained K^+^, and therefore Δ*Ψ* estimates in a less physiological buffer system would in part be confounding. Here, we calibrated the safranine‐O fluorescence signal to a known dye concentration using the Nernst equation. The amount of dye imported into the matrix was then quantified by fluorescence change relative to the depolarized FCCP state (~equivalent concentrations in the matrix and RB), this was approximately the same as baseline measurements prior to substrate addition for all temperatures.

Nernst Equation: 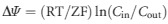


Where *R* is the gas constant, *F* is the Faraday constant, *T* is temperature in Kelvin, and *Z* is the valance state of safranine (1^+^). The safranine concentration on either side of the membrane was calculated (*C*_in_ *and C*_out_) from the change in safranine‐O fluorescence with baseline taken after the addition of FCCP. We also assumed that the mitochondrial matrix volume was fixed (2 *μ*L mg^−1^) or dynamic (2 *μ*L mg^−1^ during the Leak state and 1.2 *μ*L mg^−1^ during OXPHOS; Bazil et al. 2010), as the changes in matrix volume alter the apparent Δ*Ψ*.

We note that we have not accounted for nonspecific binding of dye to mitochondria, which is independent of Δ*Ψ* (Rottenberg 1984). However, this appears to be minimal as the fluorescence returns to near initial baseline levels following complete uncoupling. Moreover for comparative purposes the same assumptions/caveats were applied across all temperatures. We acknowledge that binding may change with temperature so present our values as an estimate of Δ*Ψ*.

### Data analysis

Data from the ex vivo working heart were collected in *LabChart* (ADInstruments) and presented as means ±SEM. Data were plotted and fitted a with segmented regression (2 piecewise) (SigmaPlot vers. 11, Systat Software, San Jose, CA) to identify break‐point temperatures (BPT) of cardiac function. BTP are reported with their standard errors and the fitted equations are plotted with 95% confidence lines shown.

Mitochondrial function data were recorded in *DatLab*5. Averaged steady‐state values for each respiratory state are presented as means ± SEM. Mitochondrial data were compared using two‐way ANOVA (SigmaPlot vers. 11). Group comparisons were made using the Holm–Sidak method. The steady‐state P:O ratio is calculated from the rate of ATP produced divided by the rate of O consumed. The RCR was calculated as OXPHOS/Leak.

## Results

### Cardiac function in the ex vivo working heart exposed to graded hyperthermia

The ex vivo working heart model was used to determine the influence of increasing temperatures on a working heart at temperatures relevant to those also tested for the isolated mitochondria. Segmented regression was then fitted to the data to determine the break‐point temperature at which function plateaus (Fig. 1A–F; Table 1). Heart rate increased linearly until 40.5°C, despite this; cardiac output steadily decreased until a break‐point temperature of 42.2°C was apparent and this contributed to a decreased stroke volume and slower rates of contraction (E) and relaxation (F). Aortic flow reaches break point at 41.0°C and showed no significant flow at 42°C.

**Table 1.** Break‐point temperatures of ex vivo working rat heart.

	Heart rate	Cardiac output	Stroke volume	Aortic flow	Max dP/dt	Min dP/dt
Break‐point temperature (°C)	40.55	42.23	41.70	40.95	40.80	41.24
Standard error	0.18	0.49	0.24	0.0077	0.43	0.37

**Figure 3.**
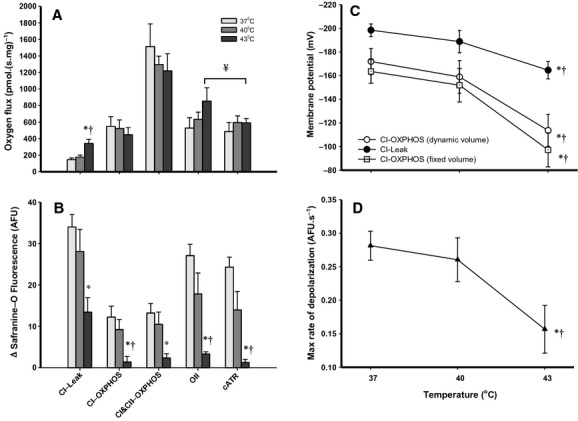
Mitochondrial respiration and simultaneous membrane potential under normothermia (37°C) and hyperthermia (40 and 43°C) (*n* = 5–6). Mean respiratory flux normalized to mitochondrial mass (A; pmolO_2._(s mg)^−1^) during different stages of the mitochondrial assay with concurrent Δ safranine‐O fluorescence (B, arbitrary fluorescence units) from after the addition of the uncoupler FCCP. CI‐Leak is initiated with GMP, oxidative phosphorylation (OXPHOS) is then stimulated by saturating concentrations of ADP and measured as CI‐OXPHOS. After the addition of succinate, CI&CII‐OXPHOS is measured. CI&CII‐Leak is then measured after the addition of oligomycin (Oli) and carboxyatractyloside (cATR). Δ*Ψ* is calculated using the Nernst equation (mV) during CI‐Leak (filled circles) and CI‐OXPHOS (C, volume corrected; open circles, fixed volume; open squares). The fluorescence transition from CI‐leak state to OXPHOS after the addition of ADP is presented as the maximum rate of change (D, filled triangles; AFU s^−1^). Significant differences between temperatures at given states are denoted by an asterisk (*) for 37°C versus 43°C (*P* < 0.05) and by a cross (†) for 40°C versus 43°C (*P* < 0.05). Significant differences between the two CI&CII‐Leak states within temperatures is denoted by ¥ (*P* < 0.01) using a paired *t*‐test.

Break‐point temperatures were determined as the intercepts of linear piecewise segmented regressions plotted in Figure 3.

### Mitochondrial bioenergetics

#### Temperature effects on mitochondrial membrane potential and respiration

A representative trace from the oxygraph and fluorometry measurements made at 37°C is presented in Figure 2. Mean O_2_ flux was measured prior to the addition of ADP (CI‐Leak) and shown to increase (by 133%) with severe hyperthermia but not mild hyperthermia (37 vs. 43°C, *P < *0.001; 40 vs. 43°C, *P =* 0.002, Fig. 3A). During all other respiration states there was no evident effect of temperature on O_2_ consumption. However, in the CI&CII‐Leak state measured after the addition of oligomycin the O_2_ flux at 43°C decreased by 31% after the addition of cATR (oli vs. oli+cATR, *P* ≤ 0.01, Fig. 3A) and this response was not seen at 37 and 40°C.

Safranine‐O fluorescence was normalized to values recorded after the addition of FCCP, therefore the fluorescence is presented as a change from the completely uncoupled, when safranine‐O fluorescence is highest in the medium (Fig. 3B). While CI‐Leak state respiration produced the highest change in safranine‐O fluorescence at 37°C, at 43°C this signal more than halved at 43°C (*P *= 0.014). No significant difference in safranine‐O fluorescence was apparent between 37 and 40°C. Following initiation of OXPHOS by ADP addition, safranine‐O fluorescence reached a new steady state, however the fluorescence change was approximately 90% lower at 43°C relative to that at 37°C (*P *=**0.008) and 40°C (*P =* 0.034). On addition of oligomycin (CI&CII‐Leak), the change in safranine‐O fluorescence decreased as Δ*Ψ* increased again, but this response was not apparent at 43°C, and even after the addition of cATR the safranine‐O signal remained unchanged. Notably the safranine‐O fluorescence did not reach the initial CI‐Leak levels in CI&CII‐Leak with oligomycin, indicating increased proton leak despite a comparative increase in respirational flux in the CI&CII‐Leak state.

The ΔΨ was calculated using a dynamic volume model to account for changes in matrix volume with transitions from Leak to OXPHOS states, where the matrix volume is predicted to contract by 40% (from 2 *μ*L mg^−1^ to 1.2 *μ*L mg^−1^, Fig. 3C) (Bazil et al. 2010). Accounting for a decreased matrix volume increases the apparent matrix safranine‐O concentration within the matrix, and this increases the calculated Δ*Ψ* by 12–20 mV. At 43°C, there was a 16% decrease in polarization of the Δ*Ψ* relative to 37°C (*P *=**0.04) in the CI‐Leak state. After the addition of ADP (CI‐OXPHOS), the Δ*Ψ* partially depolarized to a new steady state at all temperatures, however at 43°C mitochondria were 34% less polarized compared to 37°C (*P* = 0.016). We also note that the addition of succinate (CI&CII‐OXPHOS) did not alter the Δ*Ψ* significantly at any temperature despite increased O_2_ flux. The transition from CI‐Leak to OXPHOS was quantified by the maximum rate of change of safranine‐O fluorescence (Fig. 3D). The maximum transition rate was 45% slower at 43°C compared to 37°C (*P* = 0.024).

#### Temperature effects on mitochondrial ATP production and respiration

**Figure 4.**
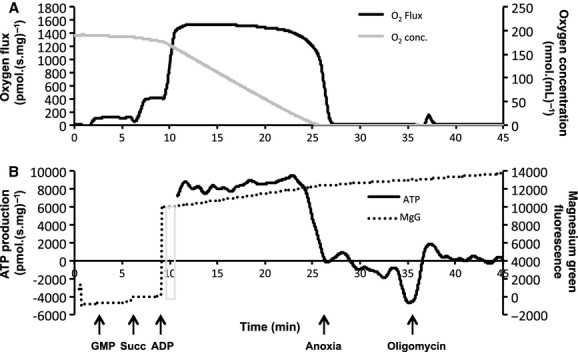
Simultaneous measurement of mitochondrial respiration and adenosine triphosphate (ATP) production. Mitochondrial respiration flux (A, black line; pmolO_2_.(s mg)^−1^) and O_2_ concentration (A, gray line; nmolO_2_ mL^−1^) is measured in one oxygraph chamber at 37°C along with magnesium green fluorescence (B, dotted line; arbitrary fluorescence units) and the corrected time derivate of MgG fluorescence which represents the real‐time ATP production rate (B, black line; pmolATP.(s mg)^−1^). Arrows show sequential titration of GMP (glutamate, malate, and pyruvate), Succ (succinate), and Mg^2+^‐free ADP. Oligomycin is added to determine zero mitochondrial ATP production and degradation. The gray bar refers to the reference point used for calibration, using the fluorescence signals prior to and after the addition of Mg^2+^‐free ADP. Sections of the MgG time derivative were omitted for clarity due to rapid shifts on addition of ADP.

**Figure 5.**
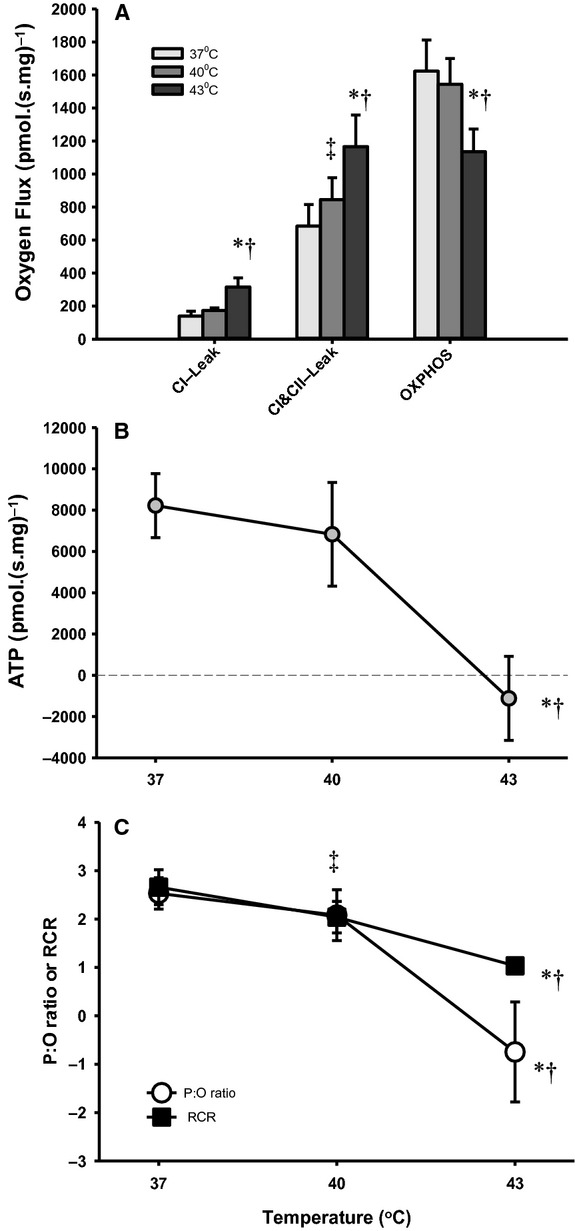
Mitochondrial respiration and simultaneous measurement of adenosine triphosphate (ATP) production under normothermia (37°C) and hyperthermia (40 and 43°C) (*n* = 7). Mean respiration flux (A, pmolO_2._(s.mg)^−1^) and ATP production during OXPHOS (B, pmolATP.(s mg)^−1^) normalized to mitochondrial mass during different respiratory steady states across all experimental temperatures. CI‐Leak is initiated by GMP and combined CI&CII‐Leak with the addition of succinate. Oxidative phosphorylation (OXPHOS) is stimulated with the addition of ADP. Mean steady‐state P:O ratio (C, white circles) and respiratory control ration (C, respiratory control ratio (RCR); black squares) with both CI and CII substrates. Significant differences between temperatures at given states are denoted by an asterisk (*) for 37°C versus 43°C (*P* < 0.05), by a cross (†) for 40°C versus 43°C (*P* < 0.05) and a double dagger (‡; RCR only in C) for 37°C versus 40°C (*P* < 0.05).

A representative trace of the respiratory and fluorometry measurements are shown for 37°C in Fig. 4. The Leak respiration O_2_ flux was measured with both CI and CII substrates (Fig. 5A) without inhibitors and was found to be 70% higher at 43°C compared to 37°C (37 vs. 43°C, *P ≤ *0.001; 40 vs. 43°C, *P = *0.005), and there was also a 23% increase at 40°C (37 vs. 40°C, *P = *0.006). Subsequent OXPHOS stimulated by the addition of ADP did not increase O_2_ flux at 43°C, but depressed flux by 30% relative to that at 37°C (*P = *0.018).

Steady‐state ATP production rates (Fig. 5B) did not differ between 37 and 40°C yet became negative at 43°C (37 vs. 43°C, *P = *0.013). When ATP production is normalized to O_2_ respiration, a steady‐state P:O ratio can be derived. In comparison to the traditional measures of OXPHOS (effective P:O ratio, or end‐point ADP/O calculations) and coupling of mitochondria (RCR), which can only reach 0 or 1, respectively, the steady‐state P:O ratio (Fig. 5C) is the only measure that enables quantification of the ATP degradation by mitochondria during hyperthermia, and this is the first time it has been reported in the presence of sufficient O_2_ (noting that the ATP synthase is reversible in anoxia; Duchen 2004).

The steady‐state P:O ratio was calculated to be 2.53 ± 0.32 at 37°C, 2.08 ± 0.52 at 40°C, and −0.75 ± 1.03 at 43°C, which highlights the negative ATP production and hydrolysis at high temperatures (37°C vs. 43°C, *P = *0.022; Fig. 5C). We note that at 43°C there was a transient positive ATP production rate after the addition of ADP, which diminished over 2–3 min before a negative steady state was reached. Therefore, at this temperature the minimal amount of ATP initially formed following ADP addition is rapidly degraded rather than being sustainably synthesized.

## Discussion

Based on our results, contractile function was maintained until temperatures exceeded ~41°C. Mitochondrial function tested with both CI and CII substrates, remains intact at 40°C (despite a slight increase in the CI&CII‐Leak state), however, between 40 and 43°C there is loss of an adequate proton gradient to maintain ATP synthesis. Moreover, the loss of Δ*Ψ* at high temperatures may then drive ATP consumption by the mitochondria during severe hyperthermia (43°C).

The ex vivo working heart showed that heart beat rate increases steadily with the onset of heating until ~41°C. We note that decreased CO and aortic flow would likely be prevented in vivo for some mammalian species, as autonomic regulation appears to sustain CO and LV contractility (max dP/dt) in dogs, which achieve maximal rates at 40°C (Sagach and Shimanskaya 1993). However, dogs appear to tolerate higher temperatures than humans and rats, as they are accustomed to storing larger amounts of heat (up to 42°C) during exercise (Chapman and Baker 1984). In the rat we noted a defined break point above 40°C, and this break‐point temperature corresponds to the threshold temperatures of 40.9°C in trained athletes, where fatigue during exercise is reported to occur (Ely et al. 2009).

### Mitochondrial function

The metabolic demands associated with hyperthermia have until now been unknown, particularly with exercise (MacDougall et al. 1974), so we compared mitochondrial bioenergetics at 37°C (normothermic) and two hyperthermic states. Our objective was to determine the effects of temperature on energy supply in the form of ATP in cardiac mitochondria when saturated with ADP (at maximum capacity). A moderate hyperthermic temperature (40°C) was used that is close to the critical temperature for exercise fatigue (Ely et al. 2009), and a critical temperature (43°C) where circulatory collapse generally occurs in mammals (Bouchama et al. 2007).

### Mitochondrial membrane leak

Energy from the flow of electrons down the ETS to O_2_ sets up the electrochemical (proton) gradient across the inner‐mitochondrial membrane (IMM). This gradient conserves energy that is then coupled to ATP synthesis at the ATP synthase (Brand 1990). In the absence of ADP it is apparent that this system is not perfectly coupled, as mitochondria respire at a low rate when no ADP is present (Leak state) and energy is lost as heat (Brand et al. 1994). In rats, up to 38% of O_2_ consumption may result from mitochondrial proton leak alone (Brand et al. 1994). Working tissues such as the heart have elevated aerobic capacities and increased fractional mitochondria masses in order produce ATP, and this translates to a high overall Leak‐mediated O_2_ flux (Brand 1990). With CI substrates alone a temperature increase from 37 to 40°C resulted in a small but nonsignificant increase in CI‐Leak O_2_ flux, while this was not associated with an increase in the Δ*Ψ* as seen by Zukiene et al. (2010), if the Δ*Ψ* decreased (Fig. 3C, NS). We did observe a significant increase in the CI‐Leak O_2_ flux at 43°C similarly seen by Zukiene et al. (2010), confirming an increase in IMM permeability. This was not surprising as increased temperature elevates membrane fluidity and induces an alteration in proton leak conductance (Park et al. 2005).

Previous studies exploring cardiac mitochondrial function during and after periods of hyperthermia (Qian et al. 2004; Zukiene et al. 2007, 2010; Nauciene et al. 2012) have only used substrates that provide single entry of electrons in the ETS (CI substrates). In vivo the ETS allows a second input of electrons via CII which should be measured during in vitro mitochondrial assays to get a more representative measure of function, however an additional substrate with entry via the electron‐transferring flavoprotein was not tested in these experiments (Lemieux et al. 2011). When CI&CII‐Leak is measured without inhibitors, as in our ATP assay (Fig. 5A), there was a moderate 23% increase in O_2_ flux at 40°C and a substantial 70% increase at 43°C compared to 37°C.

Leak respiration can increase due to the production of reactive oxygen species (ROS) at complex I; this is due to reverse electron flow from complex II (Drose and Brandt 2012). ROS increases proton conductance through the adenine nucleotide translocase (ANT), which is mediated by lipid peroxidation (Aguirre and Cadenas 2010). ANT facilitates the transport of ADP and ATP in and out of the matrix, respectively, and also contributes to basal proton leak across the IMM (Aguirre and Cadenas 2010). When CI&CII‐Leak was measured with inhibitors in the Δ*Ψ* assay there was no significant difference when measured with oligomycin or oligomycin + cATR at 37°C and 40°C. Following the addition of the ANT inhibitor (cATR) at 43°C, there was a significant drop in O_2_ flux (Fig. 3A). These data suggest that the increased Leak with hyperthermia could be partially mediated by proton flux through ANT, when respiration is fuelled by both CI and II substrates.

### Mitochondrial membrane potential (Δ*Ψ*)

While calculation of the Δ*Ψ* using a safranine‐O titration has not been employed by others, this approach resulted in comparable values reported for TPP^+^ titration in isolated liver mitochondria (Dufour et al. 1996). We lack a correction for nonspecific binding, which likely overestimates Δ*Ψ* (~25 mV; Figueira et al. 2012; Rottenberg 1984). If accounted for, the Δ*Ψ* during CI‐OXPHOS would be −144 ± 11 mV at 37°C, which is consistent with the accepted Δ*Ψ* of ~−140 mV for phosphorylating mitochondria (Zukiene et al. 2010). At 43°C this translates to a Δ*Ψ* of approximately −85 ± 13 mV during CI–OXPHOS at 43°C. Estimates of Δ*Ψ* are potentially conservative, as contraction of the matrix during OXPHOS polarized the apparent Δ*Ψ* by 12–20 mV. Yet even with matrix contraction, the Δ*Ψ* still remains below the predicted reversal potential of ANT of ~−100 mV, beyond which mitochondria no longer exchange ADP and ATP (Chinopoulos and Adam‐Vizi 2010). In addition, the Δ*Ψ* depolarization observed at 43°C may result from matrix swelling and thus our values would further overestimate Δ*Ψ* (Duchen 2004).

Following the addition of ADP, the safranine‐O dynamics were quantified during the transition from CI‐Leak to OXPHOS. This demonstrates how fast the Δ*Ψ* can respond to increased demand. We have presented the maximum rate of change in safranine fluorescence between these two states (Fig. 3D). The maximum rate of change was depressed by 45% at 43°C relative to that at 37°C, which predicts an impaired transition into phosphorylating states. The slower rate of change at 43°C may be due the initially depressed Δ*Ψ* and slower uptake of ADP, as ATP/ADP exchange requires adequate Δ*Ψ* as mentioned previously. These would depress OXPHOS initiation and the total amount of ATP that can be produced at 43°C.

### ATP production, consumption, and efficiency

ATP production and the steady‐state P:O ratio was maintained by mitochondria until limiting concentrations of O_2_ were reached at 37 and 40°C. At 43°C after the addition of ADP there was an initial positive ATP “flux” observed, although this declined to a negative steady state before the supply of O_2_ dropped (Fig. 5B). Therefore, ATP production at 43°C decreases quickly over time, and ATP starts to become degraded with adequate O_2_ supply. Heart contraction would then become dependent on a rapidly exhaustible glycolytic ATP supply alongside additional ATP consumption by the mitochondria.

Traditionally P:O ratios are determined from oxygraph recordings using end‐point protocols that extrapolate from transition states the amount of O_2_ reduced for a given amount of ADP phosphorylated to ATP (Qian et al. 2004). This approach assumes that O_2_ flux attributed to Leak remains constant and uses limiting amounts of ADP so cannot measure respiration in sustained near maximal OXPHOS states. This approach also cannot follow ATP dynamics in unusual states such as on transition from hypoxia to anoxia. Using a method adapted from Chinopolous et al. (2009), we tracked changes in free Mg^2+^ to measure ATP production in real‐time. This allowed us to calculate steady‐state P:O ratios, and revealed consumption of ATP from the respiration buffer during hyperthermia and anoxia. This information is hidden in routine measures of mitochondrial phosphorylation efficiency, such as the RCR, which can only reach 1 in completely uncoupled mitochondria (Fig. 5C).

Taken together this study demonstrates that while energy demands in the heart may increase during hyperthermia, the capacity of cardiac mitochondria to increase ATP production does not increase accordingly. Rather it is maintained at 40°C. Moreover between 40 and 43°C there is a complete collapse of OXPHOS. Our results are in accordance with published data that show increased permeability of the IMM with hyperthermia above 41°C (Nauciene et al. 2012), however we have now shown for the first time that at a temperature of 43°C there is a net consumption of ATP by mitochondria in vitro. We have also shown a decrease in CO and aortic flow with a break point at approximately 41°C. We note that with increasing temperature the mitochondrial OXPHOS and ATP synthesis capacity does not increase, while beat rate and likely ATP demand increases. Above 41°C there is a subsequent plateau in heart rate and aortic flow ceases, and this could be linked to a mismatch between a mitochondrial energy supply and demand in the heart.

Future research should look at temperatures between 40 and 43°C to better identify the precise break‐point temperature of ATP production during OXPHOS and the time it takes for irreversible changes to occur at different temperatures. In addition, it would also be important to explore the rates of ATP degradation within cardiomyocytes. As reaction rates increase with temperature, an imbalance of ATP supply and demand could contribute to the loss of cardiovascular function with more moderate hyperthermia. Assessment of mitochondrial function in situ may therefore provide further insight. Identifying the critical temperature that takes into account both the supply (which we have shown is not enhanced at 40°C) and demand for ATP in the heart may assist with hyperthermia management, such that it may be beneficial to reduce the workload of the heart in patients presenting with threatening hyperthermia.

## Acknowledgments

We would like to thank Amorita Petzer and Shorena Nachkebia for animal husbandry and tissue acquisition.

## Ethical standards

Experiments and the use of the animals for this study were approved by the University of Auckland Animal Ethics Committee and comply with the current animal welfare laws of New Zealand.

## Conflict of Interest

None declared.
